# Observational characterization of self-reported SR-17018 exposure and outcomes in individuals with prior opioid use histories

**DOI:** 10.3389/fphar.2026.1899247

**Published:** 2026-09-14

**Authors:** Mark P. Picard

**Affiliations:** Independent Researcher, Denver, CO, United States

**Keywords:** biased agonism, harm reduction, observational study, opioid dependence, opioid withdrawal, SR-17018, μ-opioid receptor

## Abstract

Opioid withdrawal remains a significant barrier to cessation, particularly in treatment-experienced populations who have made repeated unsuccessful attempts using established pharmacotherapies. SR-17018 is a μ-opioid receptor agonist with preclinical evidence of reduced β-arrestin 2 recruitment and altered receptor regulatory dynamics relative to conventional opioids. Whether these properties translate into clinically meaningful differences in humans remains unknown. This report presents a descriptive analysis of a self-reported observational registry (N = 55) characterizing SR-17018 exposure patterns, perceived outcomes, and adverse effects. Most participants (69.1%) reported three or more prior detoxification attempts, characterizing the cohort as treatment-experienced and refractory. During SR-17018 exposure, 54.5% of participants reported full cessation of opioid use and 32.7% reported meaningful reduction in use; smaller proportions reported no change (5.5%) or relapse (7.3%). Collectively, 87.3% reported improvement. These outcomes are self-reported and were not independently verified. Most participants reported dosing SR-17018 three or more times daily (76.4%), with perceived effects lasting approximately 4–8 h per dose (54.5%). Participants reported a mean withdrawal relief score of 8.20/10 (SD and distribution reported in Results), with 90.9% rating relief at seven or higher. Adverse effects were predominantly mild to moderate and included anxiety, sweating, sedation, insomnia, gastrointestinal symptoms, and subjective breathing-related concerns in a minority of respondents (7.3%); the nature of these breathing reports could not be medically verified. Because the recruitment channel selects against participants who discontinued for adverse effects or inefficacy, and because approximately half the cohort reported concurrent use of other substances, these tolerability figures—including the low discontinuation rate (1.8%) — are biased toward a favorable safety impression and cannot be read as evidence that SR-17018 is well tolerated. No objective safety monitoring was performed. These findings provide preliminary descriptive data characterizing real-world SR-17018 exposure in a treatment-experienced population. The uncontrolled, self-reported, cross-sectional design precludes any conclusion regarding efficacy or safety, and the sample is likely biased toward favorable experiences. The principal value of these data is to motivate and prioritize controlled human investigation: prospective, objectively monitored studies are required before any clinical inference about SR-17018 can be drawn.

## 1 Introduction

Opioid dependence remains a leading driver of overdose mortality and treatment burden, particularly in the context of high-potency synthetic opioids that have accelerated the complexity and severity of dependence presentations. Established pharmacotherapies—principally methadone and buprenorphine—provide effective stabilization for many individuals through sustained μ-opioid receptor (MOR) activation; however, they require long-term maintenance or gradual tapering, and a substantial proportion of treatment-seeking individuals experience repeated relapse during or following cessation attempts (Volkow et al., 2019; Mattick et al., 2009). This treatment-refractory population represents a persistent clinical challenge, and there is ongoing interest in identifying pharmacological approaches that may alter withdrawal trajectories or support cessation in individuals who have not responded to existing options. The burden of opioid use disorder extends into medically complex populations, including patients managed for cancer-related pain, where treatment decisions are further complicated by competing clinical priorities (Idrees et al., 2026).

One area of emerging interest involves biased or functionally selective MOR agonists—ligands that differentially engage downstream intracellular signaling pathways relative to conventional opioids (Schmid et al., 2017; Kenakin, 2019). The translational hypothesis underlying this framework is that ligands capable of dissociating G protein activation from β-arrestin2 recruitment may exhibit altered profiles of tolerance development, withdrawal severity, and adverse effects. However, this hypothesis has not been consistently validated in clinical contexts. Prior investigations involving atypical MOR agonists have demonstrated that preclinical signaling differences do not reliably predict improved clinical outcomes, and factors including intrinsic efficacy, receptor regulation, and pharmacokinetics substantially influence real-world drug behavior beyond simplified models of biased agonism.

SR-17018 is a MOR agonist that has been characterized preclinically as exhibiting low intrinsic efficacy for G protein activation and reduced β-arrestin2 recruitment relative to high-efficacy conventional opioids (Gillis et al., 2020; Grim et al., 2020; Bohn et al., 2000; Raehal et al., 2005). In animal models, these properties have been associated with context-dependent differences in tolerance development, antinociceptive responses, and withdrawal-related outcomes. Whether these properties translate into meaningful differences in human opioid dependence or withdrawal is not known, and no controlled clinical studies exist evaluating SR-17018 in human populations.

Despite the absence of clinical data, informal reports from harm reduction communities have described SR-17018 use in the context of opioid withdrawal management and cessation attempts. These observations are unstructured and insufficiently characterized to draw meaningful conclusions, but they represent a signal that warrants systematic description. The present study addresses this gap by analyzing data from an anonymous voluntary self-reported registry of individuals reporting SR-17018 use in the context of prior opioid exposure. Because the registry recruited from communities in which SR-17018 was already discussed, the sample is expected at the outset to over-represent individuals with favorable or noteworthy experiences; this directional bias is treated as a primary interpretive constraint throughout (see Sections 2 and 5).

The aim of this study is not to establish efficacy or clinical utility. Rather, it is to describe participant characteristics, usage patterns, temporal characteristics of SR-17018 exposure, self-reported outcomes, and adverse effects within a real-world observational cohort—thereby generating descriptive data to inform and prioritize future controlled research.

## 2 Methods

### 2.1 Study design

This study presents a descriptive analysis of data obtained from a self-reported observational registry of individuals reporting SR-17018 use in the context of opioid dependence, withdrawal, or opioid reduction attempts. The registry was designed prospectively to capture real-world exposure patterns and participant-reported outcomes in an anonymous, voluntary format. The survey instrument is publicly accessible at sr17018study.com. Data were collected between 2 April 2026 and 31 May 2026; the present analysis comprises the first 55 eligible responses received within that window, representing a cross-sectional descriptive snapshot.

### 2.2 Participants

A total of 55 participants were included in the analysis. Inclusion required: (1) self-reported history of opioid use or dependence; (2) self-reported use of SR-17018; and (3) completion of key survey fields related to substance use history and outcomes. No exclusion criteria were applied beyond incomplete or unusable responses. Participation was voluntary and no incentives were provided. All responses were anonymous; no personal identifiers were collected.

#### 2.2.1 Recruitment

Participants were recruited through an open call posted to online communities in which SR-17018 use was already an active topic of discussion, primarily Reddit communities concerned with SR-17018 and opioid harm reduction, together with SR-17018-focused discussion groups. Recruitment was open rather than systematically sampled: a link to the registry was shared within these communities, and participation depended on self-selection by members who encountered it. Secondary redistribution of the registry link by community members (snowball propagation) cannot be excluded but was not systematically tracked. No probability sampling frame existed, and the proportion of community members who saw the registry, declined, or partially completed it cannot be determined.

##### 2.2.1.1 Investigator role and conflict of interest

In the interest of full transparency, the investigator serves as a volunteer community moderator of the primary online SR-17018 discussion community from which a portion of participants was recruited. This role provided the access and credibility that made recruitment feasible, but it also constitutes a potential source of bias: the investigator is embedded in the community being studied, and participants may have been aware of the investigator’s prior interest in characterizing SR-17018 favorably. This relationship is disclosed here and in the Conflict of Interest statement, and its implications for selection and reporting bias are discussed in the Section 5.

This recruitment channel has a specific and foreseeable directional consequence: individuals who experienced positive or otherwise noteworthy outcomes with SR-17018 are more likely to participate in a community organized around the compound than individuals who tried it and discontinued for inefficacy or adverse effects. The cohort should therefore be understood as a convenience sample biased toward favorable experiences, not as a representative sample of SR-17018 users.

### 2.3 Measures

The survey captured the following domains: substance use history (primary opioid or substance; duration of opioid use, categorized; number of prior detoxification or withdrawal attempts); SR-17018 use (context of use; qualitative descriptions of dosing frequency, onset, perceived effect duration, and post-discontinuation use duration); effectiveness (withdrawal relief and craving reduction rated on 10-point scales; time to noticeable relief); outcomes (self-reported responses categorized as full cessation, meaningful reduction in use, no change, or worsening/relapse); and safety and tolerability (type and severity of adverse effects; functional impact; discontinuation due to adverse effects; concurrent substance use). Self-reported tolerance was captured as a participant-selected three-level categorical item (low/moderate/high) without formal anchoring definitions; ratings therefore reflect each participant’s own subjective assessment rather than a standardized threshold. In the concurrent-substance item, the “other opioids” option did not capture the specific agent; it indicated concurrent use of an opioid other than those separately listed. Buprenorphine and methadone were recorded under their own category and are not included in the “other opioids” group. For analysis, “any improvement” was defined as the combined proportion of participants reporting full cessation or significant reduction in use. All outcome and exposure data are based exclusively on participant self-report; no objective biomarkers, urine toxicology, medical records, or collateral-informant reports were available to verify any response.

### 2.4 Data analysis

Descriptive statistics (counts and percentages) were calculated for all categorical variables. For the withdrawal relief and craving reduction rating scales, mean, standard deviation, median, and range were calculated, both for the full cohort and within each prior-substance stratum (Section 3.6). No inferential statistical analyses were performed, consistent with the observational, self-reported, and hypothesis-generating nature of the dataset.

#### 2.4.1 Free-text coding and inter-rater reliability

Free-text responses in the adverse-effect, breathing, and discontinuation fields were categorized into predefined symptom domains using a written coding manual (Supplementary Material) that specified each category and its decision rules. Eighteen responses contained codeable symptom content and were included in the reliability assessment. The primary investigator coded all responses. To assess reliability, a second coder, blinded to the primary coding, independently categorized the same responses using only the coding manual; no attribution or causal judgments were requested of the second coder, who was instructed to code the symptom described rather than its presumed cause. Attribution (whether an effect reflected SR-17018, withdrawal, or a concurrent substance) was applied separately by the investigator per predefined rules and did not form part of the inter-rater computation.

Because responses could receive more than one symptom code (multi-label categorization), a single pooled Cohen’s κ is not well-defined; agreement was therefore assessed per category as present-versus-absent, and category prevalence is reported alongside each coefficient. The two coders were in exact agreement on all 18 responses (18/18), including complete agreement on every symptom category. Because several categories contained only a single positive instance, and both coders worked from a manual authored by the primary investigator, a chance-corrected coefficient (Cohen’s κ) is not meaningfully interpretable for these sparse categories and is not reported as a headline statistic; the agreement is presented descriptively as raw concordance on a small, hand-identified set of free-text responses. This reflects consistency in applying an investigator-defined scheme, not independent validation of the scheme itself.

Two considerations qualify the interpretation of this agreement. First, the reliability tested here is the consistency with which two coders applied an investigator-defined categorization scheme, not independent validation of the scheme itself; both coders worked from a manual, category definitions, and examples authored by the primary investigator, so the analysis speaks to independence of application rather than of the framework. Second, the assessment was performed on the subset of free-text responses containing codeable symptoms rather than on a randomly drawn sample of the full registry; a larger, randomly selected verification sample would be preferable and is a limitation of the present descriptive reliability check. The coding manual is provided as Supplementary Material.

### 2.5 Ethical considerations

This study was conducted as an independent, anonymous, voluntary, self-reported observational survey registry. As the investigator holds no institutional affiliation and therefore has no Institutional Review Board (IRB) of record, the following regulatory analysis is provided for the editor’s determination. The anonymous, identifier-free design that supports this exemption determination is also the design that precludes verification that each response represents a distinct individual; this tension between participant anonymity and data verifiability is intrinsic to the format and is acknowledged as a limitation (Section 5). The study meets the criteria for exemption under the U.S. Department of Health and Human Services Common Rule, 45 CFR 46.104(d)(2)(i), which covers research involving survey procedures in which information is recorded such that the identity of human subjects cannot readily be ascertained, directly or through identifiers linked to the subjects. This analysis was informed by the NIH Office of Extramural Research human subjects decision tool, which classified the activity as consistent with Exemption Category 2; the tool is cited as guidance and not as a substitute for institutional review.

The study met the criteria for this exemption category as follows: (1) data were collected exclusively through an anonymous voluntary online survey; (2) no names, email addresses, IP addresses, usernames, or any other direct or indirect identifiers were collected at any point; (3) responses cannot be linked to individual participants by the investigator or any third party; (4) the investigator did not contact participants, attempt to re-identify participants, or maintain any code or key capable of re-identification; (5) the study involved no intervention, no clinical contact, no administration of any substance, and no investigator-participant relationship; and (6) the research presented no more than minimal risk as defined under 45 CFR 46.102(j).

The registry was designed, built, and conducted for the sole purpose of this research, and was primary research from its first response. The survey instrument was created specifically to collect the data analyzed here; no pre-existing dataset was repurposed, and the data were not gathered for any clinical, commercial, or other non-research purpose and later adapted for study. The research purpose was disclosed to participants in the survey introduction prior to any data collection, and voluntary submission of the survey constituted implied consent to participate in research. Because the data are therefore primary research data—not a secondary use of information collected for some other purpose—and because responses were recorded anonymously and without identifiers from the outset, disclosure could not place participants at risk irrespective of the sensitivity of the subject matter. Documentation of this analysis, including the NIH decision-tool output, is available on request.

Disclaimer: This report is based on observational, self-reported data and is intended for research and informational purposes only. It does not constitute medical advice or clinical guidance. SR-17018 has not been evaluated in controlled clinical trials for safety or efficacy in humans.

## 3 Results

### 3.1 Participant characteristics

The analytic cohort comprised 55 participants (Table 1). Prior opioid exposure was heterogeneous. The single largest subgroup reported commercial 7-hydroxymitragynine (7-OH) or related products (including pseudoindoxyl and MGM derivatives) as their primary substance, representing 56.4% of the cohort (n = 31). The remaining participants reported kratom leaf or extracts (10.9%), methadone or buprenorphine (10.9%), heroin (5.5%), oxycodone (7.3%), fentanyl (3.6%), or other opioids (5.5%). No participants reported research chemical opioids as their primary substance.

**TABLE 1:** Stratified outcomes by prior substance class (N = 55).

Measure	7-OH/MGM (n = 31)	Traditional/other (n = 18)	Kratom leaf (n = 6)
Withdrawal relief — mean (SD)	8.35 (1.79)	7.83 (2.17)	8.50 (0.76)
Withdrawal relief — median; range	9; 3–10	8; 1–10	8; 8–10
Craving reduction — mean (SD)	6.32 (2.19)	7.61 (2.16)	8.00 (1.29)
Craving reduction — median; range	6; 3–10	8; 1–10	8; 6–10
Fully stopped, n (%)	18 (58.1)	10 (55.6)	2 (33.3)
Significantly reduced, n (%)	8 (25.8)	7 (38.9)	3 (50.0)
No change, n (%)	2 (6.5)	1 (5.6)	0 (0)
Relapse, n (%)	3 (9.7)	0 (0)	1 (16.7)
Any improvement, %	83.9	94.4	83.3
Subjective breathing concern, n (%)	3 (9.7)	1 (5.6)	0 (0)

All subgroup comparisons are descriptive and exploratory. The traditional/other opioid and kratom-leaf strata comprise low cell counts; estimates are statistically unstable and must not be interpreted as robust between-group differences.

Duration of prior opioid use was substantial: 70.9% reported one or more years of use, and 32.7% reported three or more years. Tolerance was predominantly high (61.8%) or moderate (36.4%). 69.1% reported three or more prior unsuccessful detoxification or withdrawal attempts, characterizing the cohort as predominantly treatment-experienced. The cohort was predominantly male (70.9%) and aged 36–45 years (48.1%). Concurrent use of other substances during SR-17018 exposure was reported by 51.9% of respondents, including benzodiazepines (25.9%), stimulants (18.5%), other opioids (14.8%), and alcohol (9.3%).

### 3.2 Self-reported outcomes

54.5% of participants reported full cessation of opioid use during SR-17018 exposure, and 32.7% reported meaningful reduction without complete discontinuation. 5.5% reported no meaningful change, and 7.3% reported relapse. Collectively, 87.3% reported some form of improvement (Table 1; Figure 1).

**FIGURE 1:**
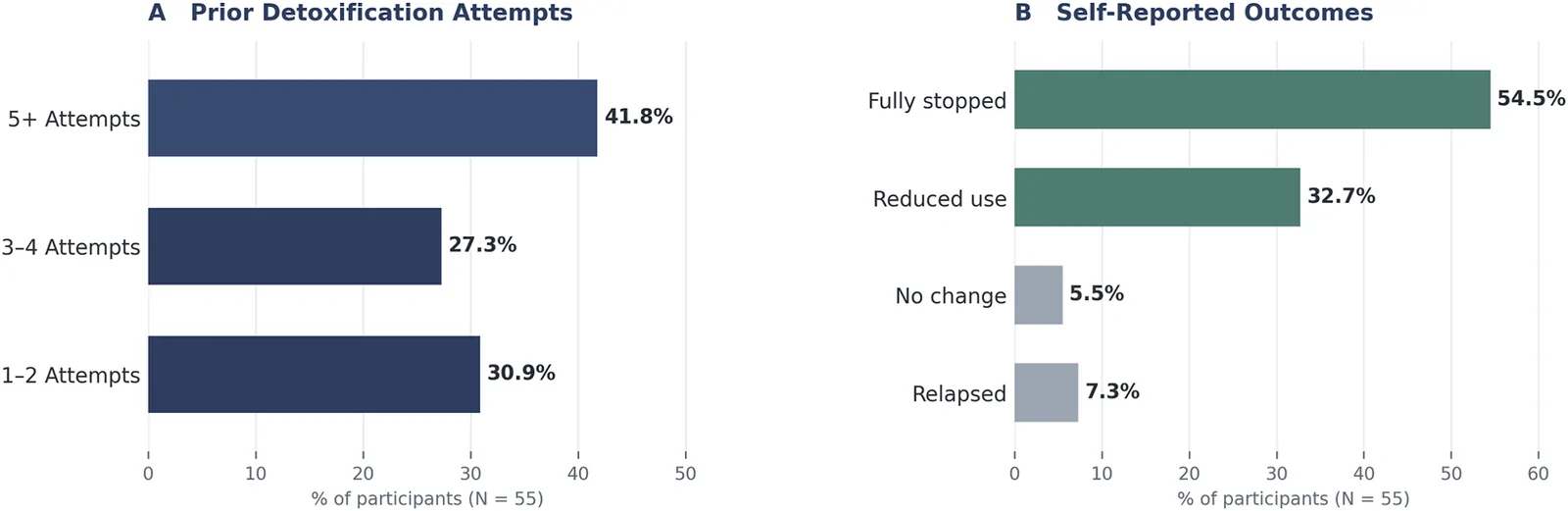
Prior treatment history and self-reported outcomes (N = 55). **(A)** Number of prior detoxification attempts; **(B)** self-reported outcome following SR-17018 use. Values represent the percentage of respondents in each category.

Participants rated withdrawal relief on a 10-point scale (1 = no relief, 10 = complete relief). The mean withdrawal relief score was 8.20 (SD 1.88; median 8; range 1–10; IQR 7–10). 90.9% rated withdrawal relief at seven or higher, and 32.7% assigned a maximum score of 10. Craving reduction was rated separately; the mean craving reduction score was 6.93 (SD 2.23; median 7; range 1–10; IQR 5–9), with 61.8% rating craving reduction at seven or higher (Figure 2). The wider dispersion of craving-reduction ratings relative to withdrawal-relief ratings (SD 2.23 vs. 1.88; IQR 5–9 vs. 7–10) accompanies the difference in their means; the divergence between withdrawal relief (8.20) and craving reduction (6.93) is described further in Section 3.6 and the discussion.

**FIGURE 2:**
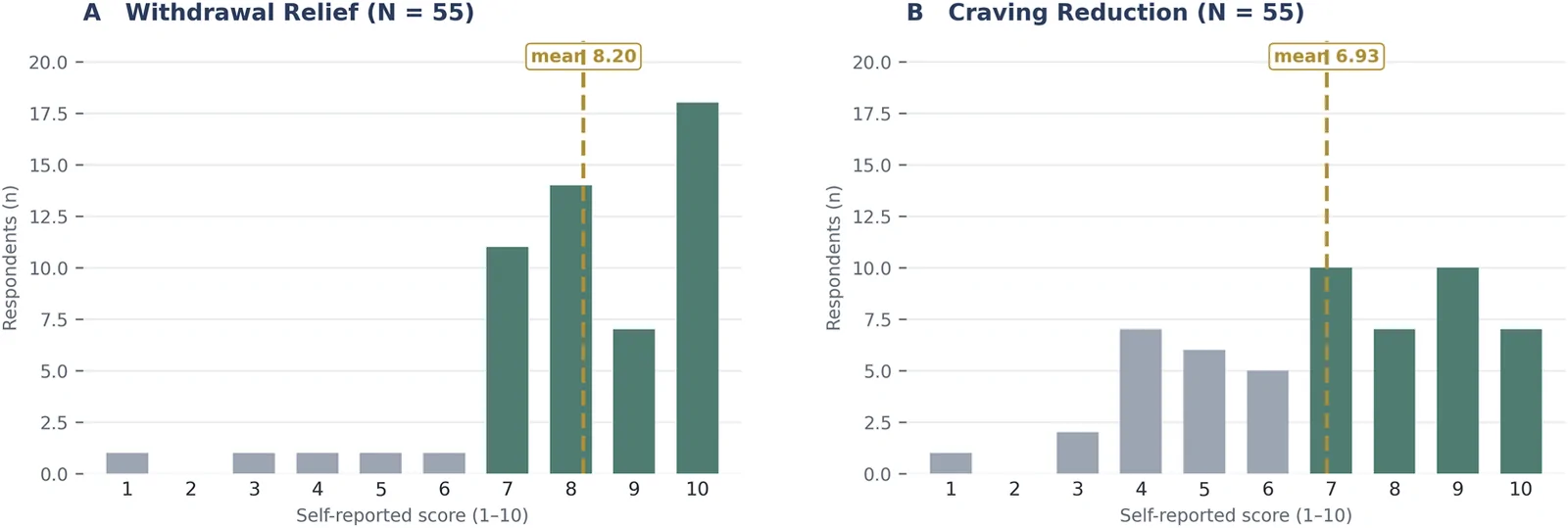
Distribution of self-reported effectiveness scores (N = 55). **(A)** Withdrawal relief (mean 8.20); **(B)** craving reduction (mean 6.93), each rated on a 1–10 scale. The dashed line indicates the cohort mean; bars at scores ≥7 are shaded to distinguish higher self-rated effect.

In qualitative responses, participants additionally described perceived reductions in withdrawal severity, decreased cravings, and improved ability to function during cessation attempts. These observations were heterogeneous and are not independently verifiable. At the time of survey completion, 34.5% reported being off all substances, while 43.6% reported still using SR-17018.

### 3.3 Temporal characteristics of SR-17018 use

76.4% of participants reported dosing three or more times daily. 70.9% reported noticeable relief within 60 min (18.2% within 30 min; 52.7% within 30–60 min). Perceived effects lasted 4–8 h per dose in 54.5% of respondents, with 27.3% reporting 8–12 h and 9.1% reporting 12 or more hours. Continued SR-17018 use following opioid discontinuation most commonly lasted 1–2 weeks (34.5%) or 4–7 days (23.6%); 29.1% reported continued use beyond 2 weeks. Collectively, 63.6% continued SR-17018 for at least 1 week following discontinuation of their prior opioid (Figure 3). Among a subset of 19 respondents who answered a question about concurrent use during taper, 73.7% (n = 14) reported using SR-17018 as an adjunct while still tapering rather than initiating it after discontinuation; this is exploratory given the limited subsample. Reported daily dosing varied widely, from approximately 10 mg to over 300 mg; participants self-sourced the compound and self-determined dosing without a standardized protocol. Per-administration dose granularity was not captured by the instrument and is noted as a measurement limitation.

**FIGURE 3:**
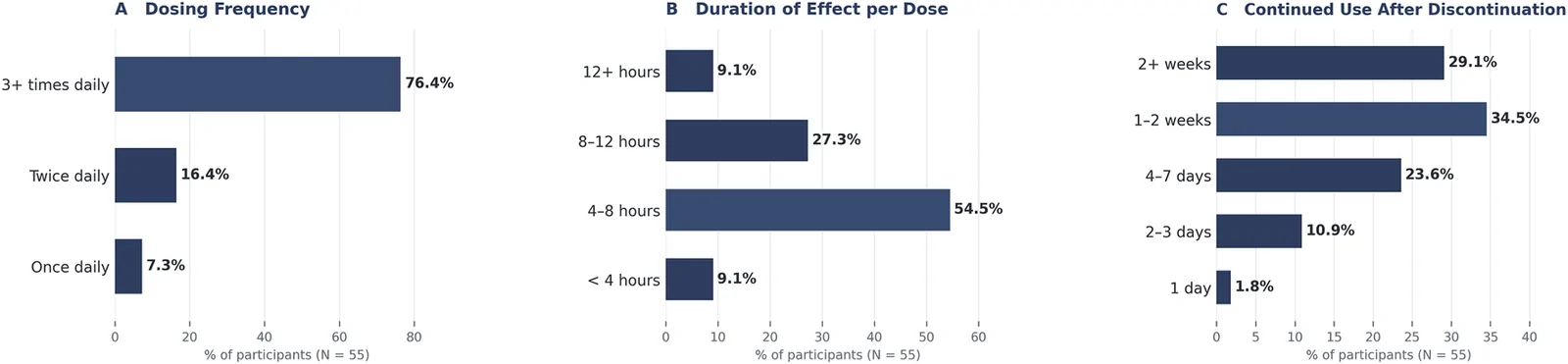
Self-reported SR-17018 use patterns (N = 55). **(A)** Dosing frequency; **(B)** duration of effect per dose; **(C)** continued use after discontinuation of the prior opioid. Values represent the percentage of respondents in each category.

### 3.4 Safety and tolerability

47.3% of participants reported no side effects on the structured severity scale, 40.0% described mild side effects, and 12.7% described moderate side effects. No participants reported severe adverse effects. Adverse effects on the multiple-selection item included anxiety (18.2%), reduced sensitivity to other opioids (20.0%), sweating (12.7%), constipation (10.9%), nausea (9.1%), headache (7.3%), insomnia (5.5%), and dizziness (3.6%). Free-text responses additionally described excessive daytime sleepiness, vivid dreams, low motivation, and anhedonia.

Sedation was rated on a 10-point scale; 49.1% assigned a rating of 1/10, indicating minimal perceived sedation in approximately half the cohort. Functional interference from side effects was reported as not present (67.3%), slight (27.3%), or moderate (5.5%); no participant reported significant functional impairment.

Four participants (7.3%) reported subjective breathing-related concerns during SR-17018 exposure. Qualitative descriptions varied: one attributed breathing changes to concurrent MGM-15 use, one described softened breathing associated with reduced fight-or-flight activation, one described intrusive pre-sleep concerns without objective compromise, and one reported occasional difficulty taking a deep breath described as hardly noticeable. The nature of these subjective reports cannot be medically verified in the absence of objective monitoring (e.g., oxygen saturation, respiratory rate, capnography). Only one participant (1.8%) reported discontinuation directly attributable to adverse effects. Interpretation of tolerability findings is complicated by the concurrent use of benzodiazepines, stimulants, and other opioids reported by approximately half of respondents.

### 3.5 Cohort composition and the attribution problem in 7-OH-predominant users

The single largest substance subgroup reported commercial 7-OH products as their primary prior opioid, representing 56.4% of participants (n = 31), with direct implications for interpretation of both outcome and tolerability findings. Commercial 7-OH products represent a pharmacologically heterogeneous exposure category that differs meaningfully from well-characterized agonists such as buprenorphine, methadone, or oxycodone. While 7-hydroxymitragynine itself is a potent MOR agonist (Kruegel et al., 2019; Obeng et al., 2021) with documented abuse liability and dependence potential, the composition of commercial 7-OH products is not standardized and often reflects synthetic enrichment inconsistent with the natural leaf (Brown et al., 2026; Alsbrook et al., 2025) and may include MGM-15 (dihydro-7-hydroxymitragynine), additional mitragynine-derived compounds, manufacturing byproducts, and other constituents of variable concentration and incompletely characterized pharmacology.

This introduces a confound distinct from the general limitations of self-reported data. Participants who used SR-17018 following prolonged exposure to commercial 7-OH products were not introducing it into a pharmacologically neutral system, but into one with extended prior exposure to opioid-active compounds of uncertain composition. When adverse effects or altered opioid responsiveness are subsequently reported, the most recently introduced compound tends to be treated as the causal agent; however, temporal association is not causation, and prior exposure to poorly characterized products is an alternative explanatory variable that cannot be excluded in an uncontrolled design.

### 3.6 Stratified subgroup analysis by prior substance class

Because the largest share of the cohort reported 7-OH/MGM products as their primary prior substance, and because these products are compositionally heterogeneous and pharmacologically distinct from conventionally characterized opioids, participants were stratified by prior substance class to examine whether aggregate findings were driven by this subgroup. Three strata were defined: (1) 7-OH/MGM products (n = 31; 56.4%); (2) traditional and other pharmaceutical or illicit opioids, comprising heroin, fentanyl, oxycodone, methadone/buprenorphine, and other unlisted pharmaceutical opioids (n = 18; 32.7%); and (3) traditional kratom leaf and leaf-based extracts (n = 6; 10.9%). Given the small size of the latter two strata, all subgroup comparisons are descriptive and exploratory; no inferential statistics were applied, and differences between strata should not be interpreted as statistically robust.

Self-reported withdrawal relief was similar across all three strata: mean 8.35 (SD 1.79; median 9; range 3–10) in the 7-OH/MGM stratum, 7.83 (SD 2.17; median 8; range 1–10) in the traditional/other opioid stratum, and 8.50 (SD 0.76; median 8; range 8–10) in the kratom leaf stratum.

Self-reported craving reduction varied more between strata: mean 6.32 (SD 2.19; median 6; range 3–10) in the 7-OH/MGM stratum, 7.61 (SD 2.16; median 8; range 1–10) in the traditional/other opioid stratum, and 8.00 (SD 1.29; median 8; range 6–10) in the kratom leaf stratum. The 7-OH/MGM stratum showed the largest divergence between withdrawal relief and craving reduction.

Outcomes were distributed as follows. In the 7-OH/MGM stratum, 18 (58.1%) reported fully stopping, 8 (25.8%) significantly reduced use, 2 (6.5%) no change, and 3 (9.7%) relapse. In the traditional/other opioid stratum, 10 (55.6%) fully stopped, 7 (38.9%) significantly reduced use, 1 (5.6%) no change, and 0 (0%) relapse. In the kratom leaf stratum, 2 (33.3%) fully stopped, 3 (50.0%) significantly reduced use, 0 no change, and 1 (16.7%) relapse. Aggregate improvement was 83.9%, 94.4%, and 83.3% across the three strata, respectively.

Subjective breathing concerns were reported by three participants (9.7%) in the 7-OH/MGM stratum, 1 (5.6%) in the traditional/other opioid stratum, and 0 in the kratom leaf stratum. The nature of these subjective reports cannot be medically verified in the absence of objective monitoring.

## 4 Discussion

This study presents a descriptive analysis of self-reported SR-17018 exposure within a real-world harm reduction population characterized by prior opioid dependence and repeated treatment failure. The central observation is that a substantial proportion of participants described perceived reductions in opioid use, withdrawal severity, or functional impairment during SR-17018 exposure. These findings are self-reported, uncontrolled, and hypothesis-generating; they do not establish efficacy. In the absence of a control group and randomization, neither placebo response nor natural fluctuation in withdrawal trajectory can be excluded as an explanation for the observed improvement. The findings represent the first structured characterization of SR-17018 use patterns and perceived outcomes in humans, and carry descriptive significance on that narrow basis alone.

The treatment history of this cohort merits attention: 69.1% reported three or more prior detoxification attempts, consistent with a treatment-refractory profile. That participants in this cohort, following multiple prior failures, described improvement during SR-17018 exposure is an observation worth characterizing, even absent a controlled comparator. It does not establish superiority over existing options; it suggests that this compound may produce subjective experiences that merit systematic investigation.

### 4.1 Stratified findings

The stratified analysis was undertaken in direct response to the concern that aggregate findings might be artifacts of the cohort’s predominance of 7-OH/MGM users. Two observations emerged, both to be read against the small size of the non-7-OH strata and the descriptive, uncontrolled nature of the data.

First, self-reported withdrawal relief was relatively consistent across all three substance strata, ranging narrowly from 7.83 to 8.50. The similarity among participants transitioning from pharmacologically diverse prior substances—including conventionally characterized full agonists such as heroin and fentanyl—suggests that the withdrawal-relief signal in the aggregate cohort is not attributable solely to the 7-OH/MGM subgroup. This pattern is consistent with, though it does not establish, a substance-agnostic mechanism of withdrawal suppression, and it cannot be interpreted as evidence of efficacy within a self-selected, self-reporting sample.

Second, the strata diverged more notably on craving reduction and relapse. The 7-OH/MGM stratum reported the lowest mean craving reduction (6.32) and the widest gap between withdrawal relief and craving reduction, whereas the traditional/other opioid stratum reported no relapses within the observation window. These differences rest on small subsamples and may reflect chance, reporting differences, or unmeasured confounding. One additional possibility warrants cautious consideration: the structural availability of the prior substance differs across strata, as 7-OH/MGM products and kratom leaf remain widely and legally available through retail outlets, whereas the prior substances of the traditional/other opioid stratum are predominantly obtained through illicit channels that may be interrupted during a cessation attempt. Persistent retail availability could plausibly sustain craving and facilitate relapse even when acute withdrawal is subjectively managed. This interpretation is speculative, is not testable within the present design, and is offered only as a hypothesis for future controlled investigation.

Taken together, the stratified findings suggest the aggregate results are not driven exclusively by the 7-OH/MGM subgroup with respect to withdrawal relief, while indicating that craving and relapse outcomes may vary by prior substance class in ways this study cannot resolve. These exploratory observations are hypothesis-generating only.

### 4.2 Mechanistic context and the craving–withdrawal divergence

The divergence between withdrawal relief (8.20) and craving reduction (6.93) at the cohort level is an observational signal that, if replicated under controlled conditions, would have mechanistic implications. A compound providing robust withdrawal suppression with more modest craving attenuation may reflect a profile distinct from full agonist maintenance agents, which typically address both dimensions through sustained receptor occupancy. Whether this reflects SR-17018’s low intrinsic efficacy, partial MOR agonism, or the limitations of self-reported scaling cannot be determined from these data. A reduction in sensitivity to other opioids was reported by 20.0% of respondents; in an uncontrolled design with 14.8% concurrent other-opioid use, this observation cannot be attributed to SR-17018 and is noted only as a candidate question for controlled study. SR-17018 has been described preclinically as a low-intrinsic-efficacy MOR agonist with reduced β-arrestin2 recruitment (Gillis et al., 2020; Grim et al., 2020); prior work shows that preclinical signaling profiles do not straightforwardly predict clinical outcomes (Schmid et al., 2017; Kenakin, 2019), and the present observations should not be read as mechanistic validation.

### 4.3 Temporal and tolerability observations

Most participants dosed three or more times daily with perceived durations of 4–8 h, and 63.6% continued SR-17018 for at least 1 week following opioid discontinuation—patterns more consistent with sustained stabilization than short-course detoxification, with implications for dosing protocols and the still-uncharacterized potential for SR-17018 dependence in humans. Rapid onset of perceived relief (70.9% within 60 min) warrants pharmacokinetic investigation. Human pharmacokinetic data on SR-17018 remain absent; the available pharmacokinetic characterization is limited to animal models, in which SR-17018 is orally bioavailable with a reported plasma half-life of approximately 6–8 h and a 3:1 brain-to-plasma distribution ratio in mice (Grim et al., 2020), alongside subsequent non-human primate work. By contrast, controlled human pharmacokinetic data are now available for the kratom alkaloids mitragynine and 7-hydroxymitragynine (Huestis et al., 2024), from which a substantial proportion of this cohort was transitioning, providing a comparator literature absent for SR-17018 itself. The compound’s receptor-level pharmacology has been characterized extensively *in vitro*, but these are pharmacodynamic rather than pharmacokinetic data, and none of this preclinical work establishes human absorption, distribution, or elimination parameters. Adverse effects were predominantly mild to moderate, with nearly half reporting none and approximately half reporting low sedation (1/10) — a potential differentiator from conventional agonists if confirmed under controlled conditions.

### 4.4 Respiratory safety

SR-17018 has been characterized preclinically as exhibiting reduced respiratory depression relative to high-efficacy MOR agonists (Gillis et al., 2020; Grim et al., 2020). The present data cannot corroborate or refute this in humans. Notably, the metabolic and receptor-level determinants of respiratory effect differ markedly across related alkaloids: 7-hydroxymitragynine produces morphine-comparable respiratory depression in animal models, whereas mitragynine does not (Hill et al., 2022; Zuarth Gonzalez et al., 2025), underscoring that respiratory safety cannot be assumed across structurally distinct MOR agonists and that unmonitored self-report cannot establish it for SR-17018. Subjective breathing-related concerns were reported by 7.3% of participants, and although no severe respiratory events or respiratory-attributable discontinuations were reported, due to the absence of any objective monitoring (oxygen saturation, respiratory rate, capnography), this study cannot make any judgment regarding the respiratory safety of SR-17018 in humans. Subjective reports in an unmonitored sample cannot distinguish benign perception from an early respiratory signal, and the preclinical reduced-depression finding must not be read as reassurance. Prospective evaluation under controlled conditions with objective respiratory monitoring is required.

### 4.5 Polypharmacy

Approximately 52% of respondents reported concurrent use of benzodiazepines, stimulants, other opioids, or alcohol during SR-17018 exposure, confounding every major outcome domain. Reported cessation may refer to the prior primary opioid rather than all substances; withdrawal relief and craving ratings may reflect the contribution of concurrent agents; and the subjective breathing reports cannot be disentangled from concurrent benzodiazepine use, which independently affects respiration. These data cannot separate SR-17018’s contribution from that of co-used substances, and outcomes should be read with that limitation foregrounded.

### 4.6 Regulatory context

On 1 July 2026, the U.S. Drug Enforcement Administration (2026) issued a notice of intent to temporarily place SR-17018 (identified as 5,6-dichloro desmethylchlorphine), together with three related benzimidazolone opioids, into Schedule I of the Controlled Substances Act under the emergency provisions of 21 U.S.C. 811(h) (Docket No. DEA-1665). The temporary scheduling order is expected to take effect no sooner than 30 days after that notice and, once effective, would impose Schedule I controls for 2 years, extendable to three. As of the present analysis, SR-17018 was not a scheduled substance, and all registry data reported here were collected prior to this action. This development is relevant in two respects. First, the DEA notice reports increasing online discussion of SR-17018 and self-administration to reduce or reset opioid tolerance. This is consistent with the real-world use context this study describes; it corroborates that the compound is used in the manner reported here, but it is an independent account of a use pattern, not verification of any pharmacological or outcome finding in this cohort. Second, Schedule I placement substantially raises the regulatory barrier to the controlled human research this study identifies as necessary, requiring specialized registration and constraining the feasibility of prospective, objectively monitored studies.

### 4.7 Research priorities

SR-17018 is demonstrably present in harm reduction environments and is being used in an uncontrolled manner by individuals with opioid dependence attempting cessation. The observational signals described here—high self-reported withdrawal relief, rapid onset, low sedation burden, and a withdrawal–craving divergence—motivate controlled human studies. Priorities include pharmacokinetic characterization; prospective safety and tolerability assessment with objective respiratory monitoring; longitudinal follow-up on cessation, relapse, and the dependence/withdrawal profile of SR-17018 itself; misuse-liability evaluation; and controlled trials examining SR-17018 and related atypical MOR agonists in opioid dependence and withdrawal management. These priorities align with broader calls for research to better inform regulation of novel opioid-active compounds (Henning et al., 2024). The 43.6% continued-use rate observed here specifically motivates longitudinal designs with defined discontinuation endpoints. Given the regulatory action described above, such studies will require Schedule I research registration; this raises, but does not eliminate, the feasibility bar for the controlled work these observations motivate.

## 5 Limitations

Several limitations substantially constrain interpretation. The dataset is entirely self-reported and subject to recall, reporting, and selection bias. As detailed in Section 2.2.1, participants were recruited through communities organized around SR-17018, and the investigator serves as a moderator of the primary such community; the sample therefore likely over-represents favorable experiences and under-captures individuals who discontinued for inefficacy or adverse effects, biasing both the improvement rate and the apparent safety profile upward. This selection pressure bears most directly on the relapse figure: a cross-sectional registry recruited from an active-user community will under-capture individuals who tried SR-17018, relapsed, and left the community, so the reported relapse rate (7.3%) should be read as a lower bound rather than an estimate, with the true rate likely higher by an unknown margin. Outcomes were not verified through any objective measure, no standardized clinical instruments (such as validated withdrawal scales like the COWS or CIWA-Ar (Wesson and Ling, 2003; Sullivan et al., 1989)) were used, and the cross-sectional design precludes causal inference. As an anonymous voluntary registry, the design also cannot verify that each response represents a distinct individual; although responses were reviewed for obvious duplication, the possibility of duplicate or repeat submissions cannot be excluded.

This is further a single-investigator, self-funded, independently run registry without institutional oversight or independent data verification. The investigator’s prior knowledge of and interest in SR-17018 cannot be fully separated from design and interpretation choices. To partially mitigate the categorization component of this risk, a blinded second coder independently categorized free-text responses with inter-rater reliability assessed (Section 2.4); however, this does not address the broader absence of institutional oversight, which remains a limitation.

Dosing patterns, exposure duration, co-administered substances, and prior opioid histories varied substantially, limiting dose-response interpretation. The 43.6% of respondents still using SR-17018 at survey completion admits multiple interpretations—mid-process stabilization versus extended use—that a cross-sectional design cannot distinguish; whether prolonged SR-17018 exposure produces dependence requiring structured discontinuation is uncharacterized in the literature.

The absence of pre-treatment baseline assessment is acknowledged; the self-anchored rating scales used are consistent with established patient-reported-outcome methodology (Stone et al., 2000; Dworkin et al., 2005; Turk et al., 2003), in which a rating reflects the participant’s own internal comparison to their pre-SR-17018 state. The predominance of commercial 7-OH users creates the attribution problem detailed in Sections 3.5, 3.6: because these products may contain MGM-15 and other constituents of uncertain activity, prior exposure histories are not fully characterizable, and effects cannot be confidently assigned to SR-17018. Whether the high improvement rate in the 7-OH-predominant subgroup partly reflects spontaneous withdrawal resolution cannot be excluded, and can be addressed only by prospective, monitored follow-up. Finally, no controlled human studies exist evaluating SR-17018’s pharmacokinetics, safety, respiratory effects, or misuse liability; these findings should be interpreted solely as preliminary observational data intended to generate research questions.

## 6 Conclusion

This study provides a preliminary descriptive characterization of self-reported SR-17018 exposure within a treatment-experienced population reporting prior opioid use histories. Participants described a range of perceived outcomes—cessation, reduction, and subjective improvements in withdrawal severity and functional stability—within a cohort marked by repeated prior cessation failure. These findings do not establish efficacy, safety, or clinical utility; they describe what a self-selected sample reported experiencing while using SR-17018 in real-world harm reduction contexts. Their value is to provide a structured descriptive foundation and specific, testable hypotheses for the controlled human studies the compound now urgently requires, moving SR-17018 from anecdote toward systematic evaluation.

## Data Availability

The original contributions presented in the study are included in the article/Supplementary Material, further inquiries can be directed to the corresponding author.

## References

[B1] Alsbrook S. Pro G. Koturbash I. (2025). From kratom to 7-hydroxymitragynine: evolution of a natural remedy into a public-health threat. Pharm. Biol. 63, 896–911. 10.1080/13880209.2025.2590311 41275505 PMC12671409

[B2] Bohn L. M. Gainetdinov R. R. Lin F. T. Lefkowitz R. J. Caron M. G. (2000). Mu-opioid receptor desensitization by β-arrestin-2 determines morphine tolerance but not dependence. Nature 408 (6813), 720–723. 10.1038/35047086 11130073

[B3] Brown P. N. Chan M. Zhang X. Brendler T. (2026). Elevated 7-hydroxymitragynine levels found in products misbranded as kratom. J. AOAC Int. 109 (1), 124–130. 10.1093/jaoacint/qsaf094 41065466 PMC12733965

[B4] Drug Enforcement Administration (2026). Schedules of controlled substances: temporary placement of 5,6-Dichloro brorphine, 5,6-Dichloro desmethylchlorphine, N-Propionitrile chlorphine, and spirochlorphine in schedule I of the controlled substances act. Notice of intent. Fed. Regist. 91 (125), 39940. Available online at: https://www.federalregister.gov/documents/2026/07/01/2026-13364/ (Accessed 05 August, 2026).

[B5] Dworkin R. H. Turk D. C. Farrar J. T. Haythornthwaite J. A. Jensen M. P. Katz N. P. (2005). Core outcome measures for chronic pain clinical trials: IMMPACT recommendations. Pain 113 (1–2), 9–19. 10.1016/j.pain.2004.09.012 15621359

[B6] Gillis A. Gondin A. B. Kliewer A. Sanchez J. Lim H. D. Alamein C. (2020). Low intrinsic efficacy for G protein activation can explain the improved side effect profiles of new opioid agonists. Sci. Signal 13 (625), eaaz3140. 10.1126/scisignal.aaz3140 32234959

[B7] Grim T. W. Acevedo-Canabal A. Bohn L. M. (2020). Toward directing opioid receptor signaling to refine opioid therapeutics. Biol. Psychiatry 87 (1), 15–21. 10.1016/j.biopsych.2019.10.020 31806082 PMC6919561

[B8] Henningfield J. E. Grundmann O. Huestis M. A. Smith K. E. (2024). Kratom safety and toxicology in the public health context: research needs to better inform regulation. Front. Pharmacol. 15, 1403140. 10.3389/fphar.2024.1403140 38887550 PMC11180979

[B9] Hill R. Kruegel A. C. Javitch J. A. Lane J. R. Canals M. (2022). The respiratory depressant effects of mitragynine are limited by its conversion to 7-OH mitragynine. Br. J. Pharmacol. 179 (14), 3875–3885. 10.1111/bph.15832 35297034 PMC9314834

[B10] Huestis M. A. Brett M. A. Bothmer J. Atallah R. (2024). Human mitragynine and 7-hydroxymitragynine pharmacokinetics after single and multiple daily doses of oral encapsulated dried kratom leaf powder. Molecules 29 (5), 984. 10.3390/molecules29050984 38474495 PMC10934259

[B11] Idrees Y. Alqaisi O. Al-Ghabeesh S. Stublla M. Sijarina L. Souied O. (2026). Opioid use disorder in cancer patients — a scoping review by experts with recommendations to improve the current situation. Explor Med. 7, 1001388. 10.37349/emed.2026.1001388

[B12] Kenakin T. (2019). Biased receptor signaling in drug discovery. Pharmacol. Rev. 71 (2), 267–315. 10.1124/pr.118.016790 30914442

[B13] Kruegel A. C. Uprety R. Grinnell S. G. Langreck C. Polepally P. R. Rice K. C. (2019). 7-Hydroxymitragynine is an active metabolite of mitragynine and a key mediator of its analgesic effects. ACS Cent. Sci. 5 (6), 992–1001. 10.1021/acscentsci.9b00141 31263758 PMC6598159

[B14] Mattick R. P. Breen C. Kimber J. Davoli M. (2009). Methadone maintenance therapy *versus* no opioid replacement therapy for opioid dependence. Cochrane Database Syst. Rev. (3), CD002209. 10.1002/14651858.CD002209.pub2 12519570

[B15] Obeng S. Wilkerson J. L. León F. Reeves M. E. Restrepo L. F. Gamez-Jimenez L. R. (2021). Pharmacological comparison of mitragynine and 7-hydroxymitragynine: *in vitro* affinity and efficacy for μ-opioid receptor and opioid-like behavioral effects in rats. J. Pharmacol. Exp. Ther. 376 (3), 410–427. 10.1124/jpet.120.000189 33384303 PMC7923387

[B16] Raehal K. M. Walker J. K. Bohn L. M. (2005). Morphine side effects in β-arrestin 2 knockout mice. J. Pharmacol. Exp. Ther. 314 (3), 1195–1201. 10.1124/jpet.105.087254 15917400

[B17] Schmid C. L. Kennedy N. M. Ross N. C. Lovell K. M. Yue Z. Morgenweck J. (2017). Bias factor and therapeutic window correlate to predict safer opioid analgesics. Cell. 171 (5), 1165–1175. 10.1016/j.cell.2017.10.035 29149605 PMC5731250

[B18] Stone A. A. Turkkan J. S. Bachrach C. A. Jobe J. B. Kurtzman H. S. Cain V. S. (2000). The Science of Self-Report. Mahwah, NJ: Lawrence Erlbaum.

[B19] Sullivan J. T. Sykora K. Schneiderman J. Naranjo C. A. Sellers E. M. (1989). Assessment of alcohol withdrawal: the revised clinical institute withdrawal assessment for alcohol scale (CIWA-Ar). Br. J. Addict. 84 (11), 1353–1357. 10.1111/j.1360-0443.1989.tb00737.x 2597811

[B20] Turk D. C. Dworkin R. H. Allen R. R. Bellamy N. Brandenburg N. Carr D. B. (2003). Core outcome domains for chronic pain clinical trials: IMMPACT recommendations. Pain 106 (3), 337–345. 10.1016/j.pain.2003.08.001 14659516

[B21] Volkow N. D. Jones E. B. Einstein E. B. Wargo E. M. (2019). Prevention and treatment of opioid misuse and addiction: a review. JAMA Psychiatry 76 (2), 208–216. 10.1001/jamapsychiatry.2018.3126 30516809

[B22] Wesson D. R. Ling W. (2003). The clinical opiate withdrawal scale (COWS). J. Psychoact. Drugs 35 (2), 253–259. 10.1080/02791072.2003.10400007 12924748

[B23] Zuarth Gonzalez J. D. Ragsdale A. K. Mukhopadhyay S. McCurdy C. R. McMahon L. R. Obeng S. (2025). Mitragynine and 7-hydroxymitragynine: bidirectional effects on breathing in rats. J. Pharmacol. Exp. Ther. 392 (11), 103720. 10.1016/j.jpet.2025.103720 41106041 PMC13169311

