# Supplementary material for: Observational characterization of self-reported SR-17018 exposure and outcomes in individuals with prior opioid use histories
Source: Front Pharmacol. 2026 Sep 14;17:1899247. doi: 10.3389/fphar.2026.1899247 (PMC13616629; doi:10.3389/fphar.2026.1899247)
Supplement: Supplementary file 1 [file Supplementaryfile1.docx]

**Free-Text Coding Manual**

SR-17018 Observational Registry — Inter-Rater Reliability Protocol

**For:** Independent second coder. **Purpose:** Independently categorize free-text survey responses by symptom so that inter-rater agreement can be assessed against the primary coder’s assignments. **Estimated time:** ~30 minutes.

**Critical instruction:** Code independently. Do NOT discuss your assignments with the primary investigator or view the primary coder’s assignments until your coding is complete and submitted. Blind, independent coding is what makes the reliability statistic valid.

# **1. Background — what you’re coding**

Participants in an anonymous survey about SR-17018 (a research opioid compound) answered open-ended questions about their experiences. Several fields contain free-text (typed) responses. Your task is to read each free-text response and assign it to one or more symptom categories using the scheme below. You do not need a pharmacology background — you are categorizing what the participant said, not judging whether it is medically accurate, and not judging what caused it.

**The free-text fields you will code:**

- **Breathing description (14a):** open text provided when a participant indicated breathing difficulty.
- **“Other” side-effect description (16a):** open text provided when a participant selected “Other” for side effects.
- **Discontinuation description (20a):** open text describing why a participant stopped SR-17018, when applicable.

# **2. How to code**

- Read the full response before assigning any code.
- Assign every symptom category that applies. A single response may receive multiple codes (e.g., a response describing both sedation and a breathing concern receives both).
- If a response clearly fits no category, assign **Z** — Uncodable/Other, and note why.
- You are coding **what symptom or effect the participant describes** — not what caused it. Do not try to judge whether an effect was due to SR-17018, withdrawal, or another substance; that determination is made separately by the investigator and is not part of your task.
- Record codes in the provided spreadsheet, one row per response, using the category letters. Separate multiple codes with commas. Do not modify the response text.

# **3. Coding categories**

*Change from prior versions: the attribution-uncertainty (U) and combination (C) modifiers have been REMOVED from the coder’s task. The second coder now codes symptoms only. Attribution judgments are applied separately by the investigator as a documented rule and are not part of the inter-rater reliability computation. Delete this amber note before sharing.*

**Respiratory / Breathing**

**R1 — Subjective breathing difficulty.** Any description of difficulty breathing, slowed, shallow, or softened breathing, trouble catching a deep breath, or air hunger. Examples: “hard to catch a deep breath,” “slowed my breathing.”

**R2 — Breathing-related fear/anxiety.** Fear, terror, or anxiety specifically about breathing or forgetting to breathe (distinct from the physical sensation itself).

**Sedation / Arousal**

**S1 — Sedation / drowsiness / sleepiness.** Feeling sedated, drowsy, sleepy, nodding off, or excessive daytime sleepiness.

**S2 — Sleep disturbance.** Insomnia, difficulty sleeping, or unusual sleep sensations/dreams (including vivid or emotional dreams, strange pre-sleep sensations).

**Energy / Mood / Motivation**

**E1 — Low energy / fatigue / exhaustion.** Descriptions of low energy, exhaustion, or fatigue.

**E2 — Anhedonia / low motivation / flat mood.** Reduced pleasure, motivation, drive, or emotional flatness. A response mentioning both low energy and low motivation receives both E1 and E2.

**Opioid Pharmacology Effects**

**O1 — Reduced sensitivity to other opioids / cross-tolerance.** Reduced response to other opioids, or a prior substance becoming less effective during SR use.

**O2 — Delayed onset of benefit.** The compound did not work initially but became effective later.

**Other Physical Effects**

**P1 — Other physical/somatic effect.** Any concrete physical effect not captured above (metabolic, appetite, blood-sugar, gastrointestinal effects described in free text).

**Catch-all**

**Z — Uncodable / Other.** Response does not fit any category above, or is too vague/ambiguous to code. Add a brief note explaining.

# **4. Ambiguous-case decision rules**

- **Multiple symptoms in one response:** code all of them. There is no limit on codes per response.
- **Vague intensifiers without a symptom** (e.g., “it was rough”): code Z if no specific symptom is named.
- **Positive/benefit statements with no adverse effect** (e.g., “worked great”): code Z unless they describe a specific codeable effect such as O2.
- **Don’t infer cause:** if a participant says “probably just withdrawal,” still code the symptom they describe (e.g., insomnia → S2). Their guess about cause does not change which symptom you code.

# **5. Worked examples**

*FABRICATED for instruction only — invented illustrations, NOT from the dataset, so none appears in the set you will code. Delete this amber note before sharing.*

| **Illustrative (invented) response** | **Code(s)** | **Reasoning** |
| --- | --- | --- |
| *"Right as I was drifting off I’d get this jolt of panic that I’d stop breathing in my sleep."* | R2 | Fear specifically about breathing. |
| *"Every so often it felt like I couldn’t pull a full breath, though it was pretty mild."* | R1 | Physical breathing difficulty. |
| *"My chest felt a little tight and my breathing seemed shallow for an hour or so."* | R1 | Physical breathing difficulty. (Any concurrent-substance detail is handled by the investigator later, not coded by you.) |
| *"Kept dozing off at my desk, and had odd half-awake sensations right before sleep."* | S1, S2 | Daytime sedation (S1) and an unusual pre-sleep sleep sensation (S2). |
| *"Really intense, emotional dreams almost every night."* | S2 | Sleep disturbance — vivid dreams. |
| *"Wiped out the whole first week, but that might just be coming off the fentanyl."* | E1 | Low energy/fatigue. Code the symptom; ignore the participant’s guess about cause. |
| *"Felt kind of flat, no interest in anything, only after being on it too long post-detox."* | E2 | Anhedonia/flat mood. |
| *"While tapering my oxy I noticed the oxy didn’t hit the same anymore."* | O1 | Reduced sensitivity to another opioid. |
| *"Nothing at all for about four days, then out of nowhere it started working."* | O2 | Delayed onset of benefit. |
| *"Wasn’t getting the usual shakes even when I ate less than normal."* | P1 | Other physical/metabolic effect. |
| *"Not sure if it was this or something else I was taking, honestly."* | Z | No specific symptom is named — the statement is only about uncertainty of cause. Code Z with a note. |
| *"Honestly it was just a rough couple of days."* | Z | Vague intensifier, no specific symptom. |
| *"Worked great, no complaints at all."* | Z | Positive statement, no codeable effect. |

# **6. Submission**

Return the completed coding spreadsheet with one row per response and the assigned symptom code letter(s) per row. Do not modify the response text. Note any cases where the manual felt genuinely ambiguous — that is useful to document. Once submitted, the primary investigator will assess agreement between your independent symptom codes and the primary coding (reported as raw concordance; a chance-corrected coefficient is not emphasized given the small, sparse category set). Your contribution will be acknowledged in the resulting publication, with your permission (named or anonymous, your choice).

*Thank you.*
